# Review and modelling of malaria crude incidence rate in a low incidence population, Illinois 1990 to 2013

**DOI:** 10.11604/pamj.2016.24.267.9904

**Published:** 2016-07-22

**Authors:** Akinyede Oyinade, Kenneth Soyemi

**Affiliations:** 1 Department of Pediatrics, John H Stroger Hospital of Cook County, 1901 W Harrison Street, Chicago, IL 60612, United States

**Keywords:** Malaria, crude incidence, Illinois

## Abstract

The highest risk of imported malaria in Illinois is associated with travel to countries of origin by immigrants to visit family and friends. We used Join point regression to analyze Malaria crude incidence rate (mCIR) trend from 1990 through 2013. We found join point regression a useful way to summarize mCIR trends because it connected the linear line segments over a fixed time interval (annual) and allowed characterization of the trends using the Annual Percent Change.

## Brief

Malaria in humans is caused by infection with one or more of several species of Plasmodium and it can be fatal if not diagnosed and treated promptly. According to the Centers for Diseases Control and Prevention (CDC), approximately 1,500-2,000 persons receive a diagnosis of malaria annually in the United States (US) [1]. Most of the reported malariacases are in travelers and immigrants (visiting family and friends(VFR)) returning from countries where malaria transmission occurs (sub-Saharan Africa and South Asia [2]. VFR travelers often have low malaria risk perception because they consider themselves immune after growing up in a malarious country. On the contrast, it is known that acquired immunity diminishes rapidly, and VFRs travelers are 8 times more likely to be receive a diagnosis of malaria when compared with tourist travelers [3].

**Figure 1:**
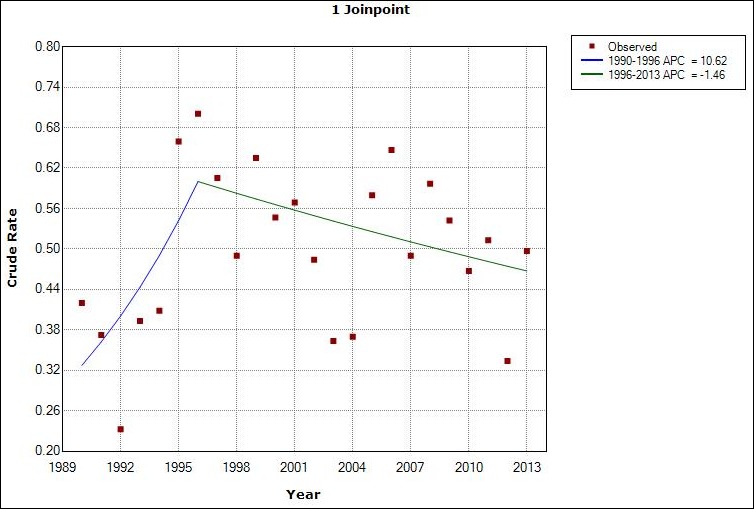
Joint point of crude malaria incidence data 1990-2013

Our study objective was to model Malaria Crude Incidence Rate (mCIR) using Join Point regression. Join point is a regression method that uses permutation tests to identify points (join points) where linear trends change significantly in direction or magnitude (e.g., zero join points indicate a straight line). The rate of change for each trend is tested to determine whether the change is significantly different from zero, and each trend in the final model is described by an Annual Percentage Change (APC) with a 95% confidence interval (CI). Join Point software computes data (e.g. disease incidence rates) trend and fits the simplest join point model that the data allow. The program starts with the minimum number of join point (e.g. 0 join points, which is a straight line) and tests whether more join points must be added to the model with the goal of detecting a statistically significant change in trend. We obtained malaria incidence data from 1990 through 2013 from the Illinois Department of Public Health (IDPH) website and calculated the mCIR using population estimate derived from census data. In the regression model, we used mCIR as the dependent variable and incidence year as the predictor variable. We used IBM SPSS Statistics for Windows, Version 22.0, and Joinpoint Regression Program, Version 4.2.0 - April 2015 for our statistical analyses. The lowest mCIR (0.2/100,000 population) was reported in 1992, and the highest (0.7/100,000 population)in 1996. There was one join point during the study period (1996). A steady increase in mCIR was observed from 1990 through 1996 with an APC of 10.6 (95% CI, = -0.4 to 22.8). From 1996 to 2013 there was a slight decrease in the mCIR APC -1.5 (95% CI, -3.4 to 0.6) (Figure 1 and Table 1).

**Table 1:** Annual percentage change

Segment	Lower End Point	Upper End point	APC	Lower CI	Upper CI	Test Statistic	P-Value
1	1990	1996	10.6	-0.4	22.8	2.0	0.10
2	1996	2013	-1.5	-3.4	0.6	-1.5	0.10

Illinois is the fifth largest state in the US with a population of over 12 million; it is estimated by the US Census that 13.8% of Illinois residents are foreign born. According to US outbound travel datafrom the National Travel and Tourism Office, in 2014, there were 30 million US travelers and three percent of these travelers listed Illinois as their state of residence [4]. Using the aforementioned data, we estimate that there will be 120,000 VFR travelers in Illinois annually with 24% travelling to Africa and Asia where malaria is endemic [5]. Also according to the Department of State Passport Office approximately 500,000 passports are issued annually to Illinois residents [6]. As worldwide travel is expected to increase, we can concurrently expect a similar increase in number of Illinois VFR travelers. When we interpolate the increased risk of malaria among VFR travelers with increased travel, we surmise that public health agencies and healthcare workers (HCWs)will continue to see imported cases of malaria in non-endemic numbers in Illinois. To mitigate infection risk, public health preventive measures to reduce mCIR should include HCWs provided education (to impending Illinois VFR travelers)regarding use of chemoprophylaxis, mosquito avoidance measures during travel to endemic areas, and HCWs should obtain travel history from all febrile patients presenting to them.

Our analysis has some limitations; mCIR was used in data analysis because of limited data available on IDPH's web site. Illinois population composition varies annually (e.g., age, gender, population composition, and net migration) making differences in mCIR difficult to interpret. We could not analyze transmission patterns and demographic trends, because we did not have access to demographic data such as age, race, and gender which are potential confounding factors.

We found join point regression a useful way to summarize mCIR trends because it connected the linear line segments over a fixed time interval (annual) and allowed characterization of the trends using the APC. Join point models of mCIR also helped us determine the slope of the incidence trend, when it started rising, peaked, and the degree to which incidence has returned to the background trend.

## References

[1] Cullen KA MK, Arguin PM (2016). Malaria surveillance-United States, 2013. Surveillance Summary.

[2] CDC (2015). Malaria Transmission Atlanta: CDC.

[3] Centers for Disease Control and Prevention (2016). Health Information for International Travel 2016..

[4] National Travel and Tourism Office (2014). Profile of U.S. Resident Travelers Visiting Overseas Destinations: 2014 Outbound..

[5] World Health Organization (2015). WORLD MALARIA REPORT 201.

[6] Department of State Passports Statistics Washington DC2015.

